# Multivariate associations of motor performance, sleep quality, depressive symptoms, and grey matter volume in younger and mid-to-older adults

**DOI:** 10.1038/s41598-025-34951-y

**Published:** 2026-01-10

**Authors:** Vincent Küppers, Hanwen Bi, Eliana Nicolaisen-Sobesky, Felix Hoffstaedter, B. T. Thomas Yeo, Alexander Drzezga, Simon B. Eickhoff, Masoud Tahmasian

**Affiliations:** 1 Department of Nuclear Medicine, Faculty of Medicine and University Hospital Cologne, University of Cologne, Cologne, Germany; 2 Institute of Neuroscience and Medicine, Brain and Behaviour (INM-7), Research Center Jülich, Jülich, Germany; 3 Institute of Systems Neuroscience, Medical Faculty and University Hospital Düsseldorf, Heinrich Heine University, Düsseldorf, Germany; 4 Centre for Sleep and Cognition & Centre for Translational MR Research, Yong Loo Lin School of Medicine, National University of Singapore, Singapore, Singapore; 5 Department of Electrical and Computer Engineering, National University of Singapore, Singapore, Singapore; 6 Institute for Health, National University of Singapore, Singapore, Singapore; 7 Integrative Sciences and Engineering Programme (ISEP), National University of Singapore, Singapore, Singapore; 8 Department of Medicine, Human Potential Translational Research Programme & Institute for Digital Medicine (WisDM), Yong Loo Lin School of Medicine, National University of Singapore, Singapore, Singapore; 9 Martinos Center for Biomedical Imaging, Massachusetts General Hospital, Charlestown, MA USA; 10 German Center for Neurodegenerative Diseases (DZNE), Bonn-Cologne, Germany; 11 Institute of Neuroscience and Medicine, Molecular Organization of the Brain (INM-2), Research Centre Jülich, Jülich, Germany

**Keywords:** Motor behavior, Sleep quality, Depressive symptoms, Mental health, Brain structure, Canonical correlation analysis, Depression, Motor cortex, Human behaviour, Computational neuroscience, Sleep, Neural circuits

## Abstract

Motor performance (MP) is essential for maintaining functional independence, particularly in later life. However, the relationship between MP and sleep quality, depressive symptoms, and their underlying brain substrates remains obscure. We employed four samples of younger/mid-to-older adults (n = 1,954) from the Human Connectome Project-Young Adult (HCP-YA), HCP-Aging (HCP-A), and enhanced Nathan Kline Institute-Rockland sample (eNKI-RS) to assess the replicability of our findings. Using canonical correlation analyses within a machine learning framework, we investigated the associations of sleep quality, depressive symptoms, and grey matter volume (GMV) with MP. In the combined model of the HCP-YA sample, a canonical variate of better sleep, mild, sub-clinical depressive symptoms, and altered GMV of several cortical (including precentral and fusiform gyrus), thalamus, and cerebellar brain regions was associated with a canonical variate of better MP (r = 0.2, SD = 0.05). This pattern was conceptually replicated in the young eNKI-RS sample (r = 0.25, SD = 0.13). In the HCP-A sample, a variate of better sleep quality, fewer depressive symptoms, and increased GMV was associated with a variate of MP (r = 0.18, SD = 0.1), but these findings did not replicate in the mid-to-older eNKI-RS sample (r = 0, SD = 0.12). Across all samples, variates of increased GMV were associated with variates of better MP, suggesting potential neuroanatomical underpinnings. We observed age-related variations in the multivariate associations between sleep quality, depressive symptoms, and GMV with MP.

**Supplementary Information:**

The online version contains supplementary material available at 10.1038/s41598-025-34951-y.

## Introduction

Motor performance (MP), including dexterity, strength, endurance, gait, and processing speed, is fundamental to human functioning. Impairment of MP is linked with functional dependency, particularly in older adults, reduced quality of life and well-being of the general population and patients with neurological and psychiatric disorders^1,2^and increased overall mortality^3^. However, what are the psychological and neural contributing factors to MP in the general population?

Previous studies have demonstrated the role of various demographic, physical, psychosocial, and lifestyle factors on MP^4,5^. Among them, poor sleep and depressive phenotype are highly prevalent nowadays^6,7^ and strongly interrelated^8,9^. Sleep disturbances have been shown to serve as a predictor for depressive symptom severity, and individuals suffering from insomnia are twice as likely to develop future depression. In turn, most individuals diagnosed with depression report experiencing sleep complaints. Furthermore, sleep disturbances are a prevalent residual symptom that persists following treatment^8–12^. Both aspects adversely affect physical and mental health^13–15^ and have been suggested as potential contributors to abnormal MP^16^. For example, acute sleep loss affects the MP of athletes, leading to reduced performance the following day^17^. Several pieces of evidence point to potential links between muscle strength and sleep duration^16,18^, although the results remained inconclusive in a meta-analysis^19^. In addition, decreased depressive symptoms are associated with higher MP, such as grip strength and cardiovascular fitness, in the general adult population^20–22^. Thus, it is crucial to assess the role of both sleep disturbance and depressive symptoms on MP, which are frequent everyday stressors in the general population and precursors for developing clinical conditions^23^. By investigating these factors within the general population, we aimed to identify how subclinical sleep disturbance and depressive symptoms are linked to MP, thereby pointing to early opportunities for preserving function and preventing later MP decline. Additionally, both sleep disturbances and depressive symptoms co-occur in chronic clinical conditions involving motor dysfunction, including neurodevelopmental disorders, major depressive disorder (MDD), bipolar disorder, schizophrenia, Parkinson’s disease, Alzheimer’s disease, and stroke^24–28^. Hence, understanding their interplay with MP may inform the development of novel preventive strategies and therapeutic approaches in the future.

Neuroimaging studies identified brain structure correlates (mainly grey matter volume (GMV)) with MP across various cortical, subcortical, and cerebellar regions^29,30^. Increased GMV in frontal and parietal areas has been observed among physically active adults^31^. Furthermore, grip strength is associated with GMV variations in subcortical, limbic, and temporal regions^32^. However, the relationship between sleep quality and depressive symptoms with regional GMV remains inconclusive. For example, large-scale studies identified a link between longer sleep duration and higher GMV in the basal ganglia but have failed to establish significant associations between other sleep health dimensions and GMV, such as insomnia^33,34^. Similarly, neuroimaging investigations on depressive symptoms revealed inconclusive findings, with an earlier large-scale, multi-cohort study identifying small reductions in the hippocampal volume in patients with MDD^35^. Recent large-scale studies found replicable GMV differences in regions such as the insula, thalamus, orbitofrontal cortex, and fusiform gyrus between patients with MDD and healthy controls^36^. However, these effects are small and currently do not permit generalizable prediction of disease status from brain structure^8,37,38^. Equally, the evidence for shared neuroanatomical links between sleep disturbances and depression remains inconclusive. However, recent work has highlighted the involvement of the insula, anterior cingulate cortex, thalamus, the orbitofrontal cortex, and the salience network^39^. A large-scale study reported shared associations between insomnia and depressive symptoms and a smaller thalamus and reduced total cortical surface area^40^. Therefore, the neurobiological correlates of the interplay between sleep quality and depressive symptoms with MP remain unclear.

Ageing shows notable influence on motor function (e.g., slower movement speed and coordination^30,41^), sleep patterns (e.g., more fragmented sleep patterns and worse sleep quality^42^), depressive symptomatology and related factors (e.g., loneliness^43,44^), and atrophy in regional and global GMV^45^. Considering these omnipresent age-related variations within the mentioned domains, we conducted separate analyses for younger and mid-to-older adults to assess the differential impact of ageing on the multivariate association between these domains. Evidence suggests that individual variability in motor ability, physical activity, and related neural markers is already present in early adulthood and may shape long-term trajectories of motor and cognitive function^46–49^. The inclusion of younger adults offers potential insights into early-life factors that may influence optimal MP in athletes, as well as vulnerability or resilience to age-related MP decline. Furthermore, we hypothesized that 1) stronger MP is associated with better sleep quality and fewer depressive symptoms, and this relationship is anchored in measurable parameters of macroscale brain structure; 2) a combination of sleep quality, depressive symptoms, and GMV has the strongest association with MP; and 3) such multivariate associations are different between younger and mid-to-older adults. Four samples from three independent publicly available cohort studies were used to assess the replicability of our findings. We calculated regularized canonical correlation analyses (rCCA) to explore the link between individual domains (i.e., sleep, depressive symptoms, GMV), and their combinations in association with various MP domains (i.e., dexterity, strength, endurance, and processing speed). Such multivariate association methods enable us to assess bidirectional associations between at least two sets of variables, identifying their linear combinations by maximizing the correlation between them. Thus, rCCA is particularly helpful when studying complex interconnected domains, reduces Type I error, increases the statistical power of variables, and reveals latent patterns missed by univariate methods^9,50,51^. Hereinafter, behavioral contributing factors of MP, represented by sleep quality and depressive symptoms, were used as behavioral domains to distinguish phenotypic measures from neuroimaging variables.

## Methods

### Participants and phenotypic data

We used data from 1,954 individuals across three openly available imaging cohorts (the Human Connectome Project Young-Adult (HCP-YA), the Human Connectome Project Aging (HCP-A), and the enhanced Nathan Kline Institute-Rockland Sample (eNKI-RS)), and split them into two samples of younger adults and two samples of mid-to-older adults (Table 1)^52–54^. Sleep quality was measured with the Pittsburgh Sleep Quality Index (PSQI)^55^, depressive symptoms were assessed with depression-related items from the Adult Self Report (ASR)^56^, and MP was evaluated by different motor tasks that varied across the cohorts^52,53^ (see Table 1 and Supplementary methods and Supplementary Figure S9-S11 for details). Group differences in younger and mid-to-older adults were assessed using Mann–Whitney U tests for PSQI and ASR scores and Welch’s t-test for approximately normally distributed MP items. Effect sizes (rank-biserial correlations, Mann–Whitney U tests) showed very small differences in sleep quality between HCP-YA and HCP-A (r = 0.07), and no significant differences in the eNKI-RS Young and eNKI-RS Old (r = −0.01), while for depressive symptoms, a small difference was observed between HCP-YA and HCP-A (r = 0.23) and no difference between the eNKI-RS Young and eNKI-RS Old (r = 0.02). Comparisons of MP items showed higher differences between younger and mid-to-older adults with effect sizes (Cohen’s d for t-tests) ranging from moderate effect sizes in gait speed (HCP-YA vs. HCP-A: d = 0.25) to substantial differences in processing speed (HCP-YA vs. HCP-A: d = 1.28; eNKI-RS Young vs. eNKI-RS Old: d = 1.5) (see Table 1 for all statistical comparisons).

**Table 1:** Sample characteristics and phenotypical assessments. Data distributions of the items are shown in Supplementary Figures S9-S11. Group differences between younger and mid-to-older adults were assessed using Welch’s t-test for approximately normally distributed items and Mann-Whitney U test for non-normally distributed items. HCP-YA, Human Connectome Project Young Adult; HCP-A, Human Connectome Project Aging; eNKI-RS, enhanced Nathan Kline Institute Rockland Sample; PSQI, Pittsburgh Sleep Quality Index; ASR, Items of the (Old) Adult Self Report questionnaire associated with depressive disorder, without sleep-related questions; MP, motor performance; NIH, assessments from the NIH Toolbox for motor and cognition; CNB, Penn Computerized Neurobehavioral Battery; TMT, Trail Making Task (from Delis-Kaplan Executive Function System).*Sum score of eleven items of the Depressive Problems ASR DSM-oriented scale, excluding items unavailable in ASR for older adults (OASR) and sleep-related items (ASR 24, 77, 100), maximum score = 22.

	**HCP-YA**	**HCP-A**	**eNKI-RS Young**	**eNKI-RS Old**	**HCP-YA vs. HCP-A**	**eNKI-RS Young vs. eNKI-RS Old**
Sample size	1086	358	230	280	-	-
Female participants	587	200	128	199	-	-
Age range, years	22–37	50–85	18–40	50–85	-	-
Age, mean (SD), years	28.8 (3.7)	65.1 (9.9)	26.7 (6.3)	63.3 (8.4)	-	-
Sleep quality (PSQI), mean (SD), total score	4.78 (2.76)	4.4 (2.61)	4.92 (2.72)	5.14 (3.26)	U = 207,986.00, p = 0.045, r = 0.07	U = 31,841.00, p = 0.828, r = −0.01
Depressive symptoms (ASR), mean (SD), raw total score*	2.44 (2.76)	1.51 (2.17)	2.07 (2.76)	1.97 (2.67)	U = 238,231.00, p <.001, r = 0.23	U = 32,892.00, p = 0.665, r = 0.02
MP: Strength	Grip strength (NIH)	Grip strength (NIH)	Grip strength	Grip strength	t(627) = 25.85, p <.001, d = 1.55	t(446) = 5.69, p <.001, d = 0.52
MP: Endurance	2-Minute walk test (NIH)	2-Minute Walk Test (NIH)	-	-	t(571) = 15.28, p <.001, d = 0.97	-
MP: Cardiorespiratory fitness	-	-	Bike – VO2max	Bike – VO2max	-	t(t(486) = −3.19, p = 0.002, d = −0.28
MP: Gait speed	4-Meter walk gait speed test (NIH)	4-Meter walk gait speed test (NIH)	-	-	t(507) = 3.64, p <.001, d = 0.25	-
MP: Dexterity	-	-	Grooved pegboard	Grooved pegboard	-	t(407) = 11.10, p <.001, d = 0.93
MP: Processing speed	Pattern comparison processing speed test (NIH)	Pattern comparison processing speed test (NIH)	Mouse practice task (CNB)	Mouse practice task (CNB)	t(588) = 20.59, p <.001, d = 1.28	t(464) = 17.68, p <.001, d = 1.50
MP: Motor speed	-	-	TMT-motor speed	TMT-Motor speed	-	t(506) = 7.24, p <.001, d = 0.63

Higher PSQI scores in each variable indicate worse sleep quality, while higher ASR scores indicate more depressive symptoms. Conversely, higher MP scores indicate better performance. We combined the HCP-YA and HCP-A to identify potential age group-independent effects based on the common motor tasks. Combining all three cohorts was impractical due to the different motor tasks across the cohorts. The Ethics Committee of Heinrich Heine University Düsseldorf approved re-analyzing data from the different cohorts (Approval No. 4039). All participants involved in the original data collection (HCP and eNKI-RS) provided informed consent. All methods were performed in accordance with the relevant guidelines and regulations.

### MRI pre-processing & GMV analysis

T1-weighted scans of all cohorts were pre-processed using the Computational Anatomy Toolbox Version 12.8.2 (CAT12)^57^ (see Supplementary material for details). The mean GMV for each participant was estimated in native space, as implemented in the region of interest analysis by CAT12. A total of 262 regions covering the whole brain were calculated using the Schaefer atlas (200 cortical areas), the Melbourne subcortex atlas (scale II), and the spatially unbiased atlas template of the cerebellum and brainstem (SUIT)^58–60^. The images underwent an automatic quality control by CAT12; all scans with an image quality rating (IQR) of > 3.5 were excluded.

### Regularized canonical correlation analysis (rCCA)

**Fig. 1:**
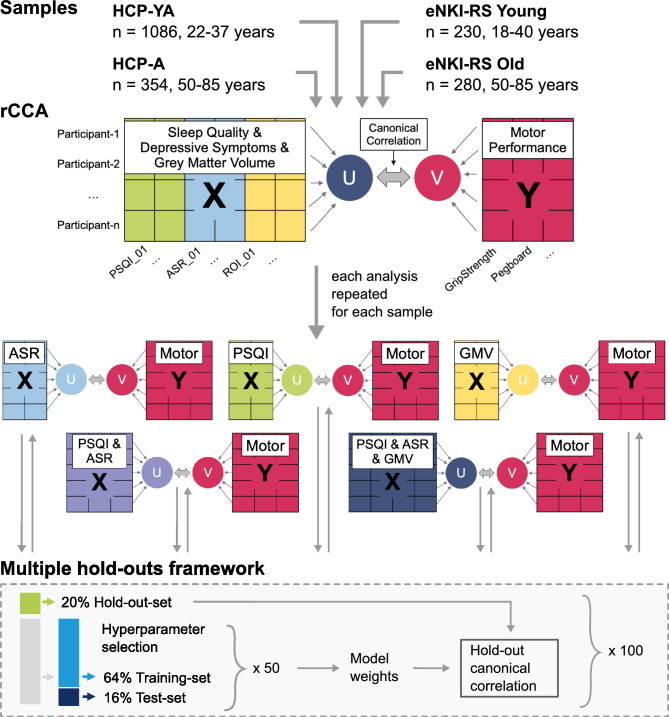
Flowchart of the analysis pipeline. Three cohorts were employed, comprising two samples of young (18–40 years) and two samples of mid-to-older (50–85 years) participants. For each sample, five different regularized canonical correlation analyses (rCCA) for individual and combined domains were computed and tested in a machine learning framework. HCP-YA, Human Connectome Project Young Adult; HCP-A, Human Connectome Project Aging; eNKI-RS, enhanced Nathan Kline Institute Rockland Sample; PSQI, Pittsburgh Sleep Quality Index; ASR, Items of the Adult Self Report questionnaire associated with depressive disorder; GMV, Grey matter volume.

To uncover the multivariate relationship between sleep quality, depressive symptoms, and brain structure with MP, we computed five different regularized canonical correlation analyses (rCCA) for each sample in the main analysis (Fig. 1). The CCA assesses the links between two sets of variables by identifying the linear combinations of variables within each set, such that the resulting linear combinations are maximally correlated between sets. In this context, a *canonical variate* refers to a specific linear combination, and a *mode* represents a pair of these linear combinations, one from each variable set, that are maximally correlated. We used a regularized version with L2 penalty function to address the high dimensionality and potential multicollinearity among the large number of variables, particularly from the GMV variables. The L2 regularization stabilizes the results by penalizing the magnitude of the coefficients, thereby reducing the risk of overfitting and the impact of multicollinearity^51,61^. To control for potential confounding factors, we regressed out age, age squared, and sex in all rCCA models in a cross-validation consistent manner, avoiding potential data leakage^62^. Additionally, to ensure that the findings were not biased by variation in head size, the total intracranial volume was regressed out for the rCCA models that included GMV. The cohorts’ effect was included as a confounding factor in the supplementary analysis using the combined HCP-YA and HCP-A datasets.

In the present study, domain X included either (1) depressive symptoms, (2) sleep quality, (3) brain structure, (4) a combination of depressive symptoms and sleep quality, or (5) a combination of depressive symptoms, sleep quality, and GMV. Domain Y included MP variables. The pairs of canonical variates describing the relationship between each domain X and domain Y are computed and tested for stability and generalizability using a machine learning framework that uses multiple hold-outs of the data (see following section) (Fig. 1). Here, we only presented the first mode of association, as all other modes showed low generalization to the hold-out data (Supplementary Figure S1).

We calculated canonical loadings to interpret the modes of the rCCA models. These are Pearson correlations of the input variables with the canonical variates calculated in rCCA and can be interpreted as factor loadings. These loadings indicate the extent to which each input variable contributes to the multivariate association between the variables across the two domains (X and Y). It is important to note that the canonical correlations themselves reflect the association between the derived canonical variates (the multivariate composites), rather than direct associations between the original input variables. For instance, a positive canonical correlation does not directly imply that more depressive symptoms are associated with better MP at the univariate level. Instead, it reflects the shared variance between the broader multivariate patterns across domains. Interpretations about specific variables and the direction of their relationship should therefore be based on inspecting the canonical loadings within each domain.

In addition to canonical correlations and loadings, *redundancy indices* were calculated as an additional metric for model performance. The redundancy indices for the first mode of Y were computed to quantify the proportion of variance in the variables of Y that can be explained by the canonical variate derived from X. This is done by computing the canonical loadings for Y, squaring these loadings, and averaging them. This amount of shared variance is multiplied by the coefficient of determination from the canonical correlation (i.e., the squared canonical correlation) to obtain the redundancy index. Thus, the redundancy index quantifies the extent to which variance from one domain of variables can be meaningfully explained by the canonical association with the other domain.

### Robustness and generalizability of rCCA models

We applied a multiple hold-out framework to assess the robustness of our rCCA models^51^. Specifically, we divided the data into a 20% hold-out set and an 80% optimization set (i.e., outer split). The optimization set was then further divided into 80% for training and 20% for testing (i.e., inner split)^50^. The optimal rCCA hyperparameters were estimated by computing the Euclidean distance in a two-dimensional space of the stability, the similarity of weights, and the canonical correlation coefficients, of the measured vs. the perfect stability and correlation^63^. This was done in the inner split and repeated 50 times. Subsequently, the hold-out set was projected onto the weights of the optimal rCCA model obtained from the inner split to estimate the hold-out canonical correlation. To ensure the robustness of the found associations, the outer split was randomly repeated 100 times. The hold-out correlations and canonical loadings reported in our analyses represent averages across all 100 repeats. To consider the family structure within the HCP-YA, members of the same family were kept in the same data split, as implemented using the exchangeability blocks within the CCA/PLS toolkit^8,51^. The hold-out canonical correlations are a measure of the extent to which the canonical association identified in the optimization set generalizes to unseen data, thereby serving as an estimate of the model’s robustness. Hold-out correlations are canonical correlations and should therefore be interpreted as reflecting the multivariate association between the derived canonical variates from each domain, rather than direct association between the original data.

### Statistical significance testing of rCCA models

The statistical significance of the hold-out canonical correlations was assessed using a permutation test with 5000 repeats. The 100 previously computed outer splits were used, and the regularization parameters were fixed to a single pair equal to the median of the per-split values. The null distribution was generated by permuting the rows of Y across all participants. For each permutation, we drew once and applied this mapping consistently across all outer folds. Within each outer fold, the training pipeline was run on the permuted training set. The hold-out set was projected onto the rCCA weights, and the hold-out canonical correlation was computed. The hold-out-validated summary statistic was the Fisher z mean across the 100 hold-out correlations. The Fisher Z-transformation was performed to derive normally distributed variables, which could then be used for statistical testing. The statistical significance was determined by computing a one-sided p-value as the proportion of permutation statistics that were at least as large as the observed value. It should be noted that the statistical significance of this test does not necessarily imply its generalizability. At best, it indicates whether the observed associations exceed what would be expected by chance under the permutation scheme.

Hold-out correlations of the 100 outer splits were compared to facilitate comparisons between rCCA models within a sample across different domains. The canonical correlation coefficients of each domain and sample were Fisher z-transformed. Pairwise comparisons were computed between all domains within one sample. Given the interdependence between the optimization and hold-out sets across repetitions, variance may be overestimated^64^. Therefore, we corrected the t-tests^65^. P-values for all comparisons within one rCCA model are corrected using False Discovery Rate (FDR) and p < 0.05 is considered as a significant difference between rCCA models. We additionally compared the redundancy indices of Y.

### Cross-cohorts replicability

To address the importance of replicating results in neuroscience, we performed cross-cohort comparisons to ensure the replicability of our findings through qualitative replication^66^. The models calculated in this study use the same questionnaires for sleep quality and depressive symptoms, as well as the same calculations for GMV. Spearman correlations were computed separately to compare the rCCA models between the averaged loadings of (1) GMV and (2) PSQI and ASR. However, as the measures of MP differed, the comparisons remained conceptual. The first mode of each sample was compared with the others, and p-values were corrected for multiple comparisons using FDR correction.

## Results

**Fig. 2:**
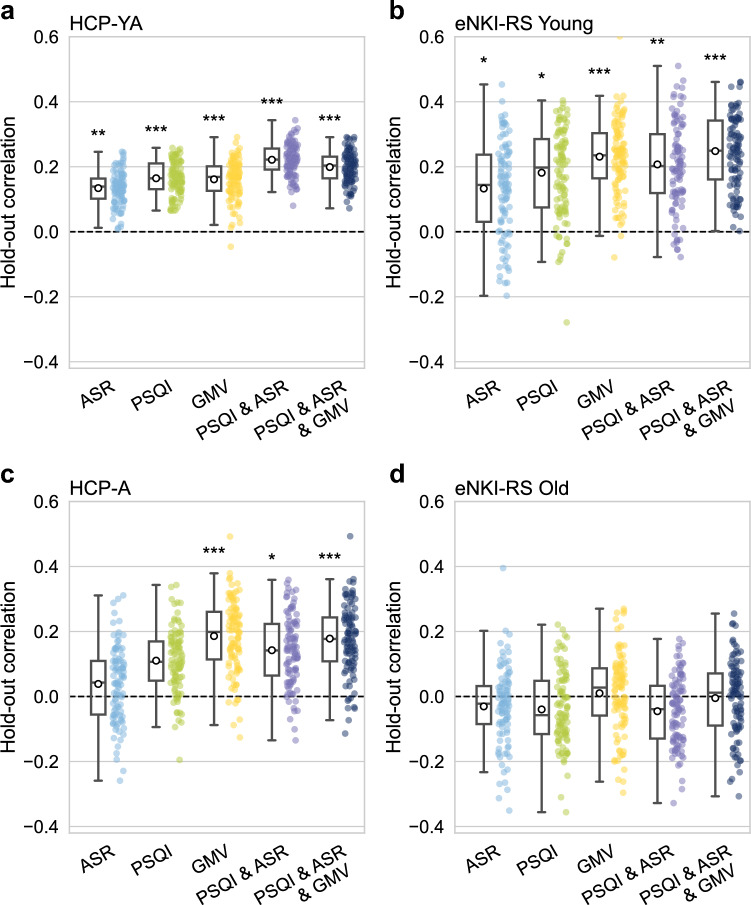
Individual and combined regularized Canonical Correlation Analysis (rCCA) results across four samples. Multivariate associations between depressive symptoms (ASR), sleep quality (PSQI), grey matter volume (GMV), and their combinations with measures of motor performance. Canonical correlation values correspond to the hold-out canonical correlations, computed in 100 outer splits for every model, and in each sample.

Consistent and replicable multivariate associations of depressive symptoms, sleep quality, and GMV with MP were observed in the HCP-YA, eNKI-RS Young, and HCP-A samples. Conversely, the eNKI-RS Old sample failed to generalize to the hold-out set in all rCCA models, reflecting more variability. Mild canonical correlations were observed between depressive symptoms and MP, with average hold-out correlations of 0.13, SD = 0.05 (HCP-YA), 0.13, SD = 0.15 (eNKI-RS Young), 0.04, SD = 0.12 (HCP-A). Sleep quality showed slightly stronger canonical correlations with MP, yielding hold-out correlations of 0.17, SD = 0.05 (HCP-YA), 0.18, SD = 0.14 (eNKI-RS Young), 0.11, SD = 0.1 (HCP-A). GMV demonstrated associations with MP across three samples, with hold-out correlations of 0.16, SD = 0.06 (HCP-YA), 0.23, SD = 0.12 (eNKI-RS Young), 0.19, SD = 0.12 (HCP-A) (Fig. 2a-d).

When our behavioral domains (sleep quality and depressive symptoms) were combined into a single rCCA model, a stronger multivariate association with MP was observed compared to the individual models of PSQI and ASR. In the HCP-YA sample, a hold-out correlation of 0.22, SD = 0.05, was observed, as well as in the eNKI-RS Young with 0.21, SD = 0.14, and in the HCP-A sample, with 0.14, SD = 0.11. The models combining PSQI, ASR, and GMV showed correlations with MP of 0.2, SD = 0.05 (HCP-YA), 0.25, SD = 0.13 (eNKI-RS Young), 0.18, SD = 0.1 (HCP-A), and 0.22, SD = 0.05 (HCP-YA and HCP-A, Supplementary Figure S2a). These combined models in the HCP-YA consistently yielded stronger associations than individual models (Fig. 2a-d) and showed higher redundancy index scores (Supplementary Figure S3a), indicating that the proportion of the variance of MP explained by the canonical variate derived from the combined model is greater than that explained by the individual domain models. While visual inspections suggested differences between models, formal comparisons using corrected resampled t-tests (FDR corrected for multiple comparisons) showed only for the comparison in the HCP-YA between the ASR vs. MP and ASR & PSQI vs. MP models statistical significance (canonical correlations: t = −5.34, p < 0.001; redundancy indices: t = −4.41, p < 0.001).

### Canonical loadings in young adults

**Fig. 3:**
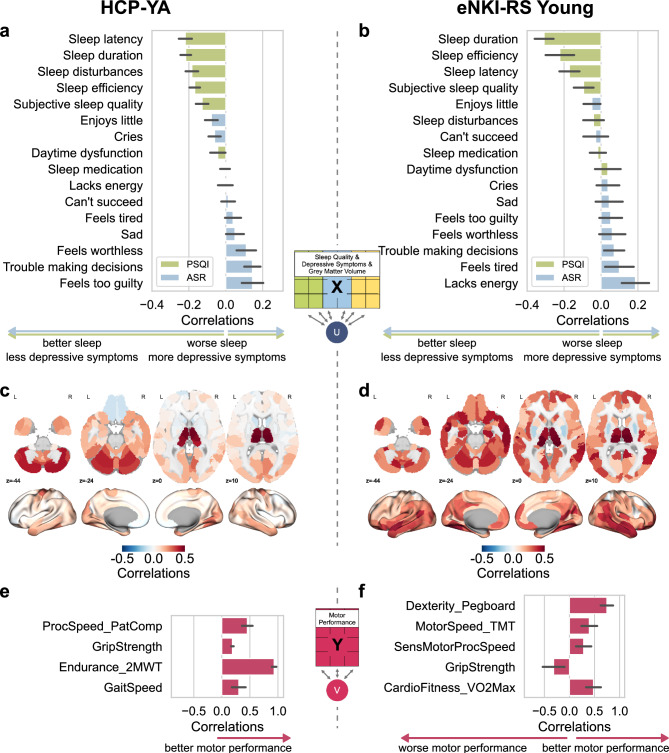
Loadings of regularized Canonical Correlation Analysis of the model combining PSQI, ASR, and GMV vs. motor performance in two samples of young adults. a, b, c, d, All variables of sleep quality (PSQI – Pittsburgh Sleep Quality Index), depressive symptoms (ASR – Adult Self Report), and grey matter volume parcels are correlated with the canonical variate U; Negative loadings of PSQI and ASR indicate better sleep quality, and less depressive symptoms, while positive loadings indicate worse sleep quality and more depressive symptoms; e, f, All variables of motor performance (Pegboard: Grooved Pegboard (eNKI-RS); PatComp: Pattern comparison task from NIH toolbox (NIH); SensMotorProcSpeed: Sensorimotor processing speed – Mouse Practice Task from Penn Computerized Neurobehavioral Battery; TMT: Trail Making task (from Delis-Kaplan Executive Function System); 2MWT: 2-Minute Walk Test (NIH); VO2Max: Cardiovascular fitness estimated from bike test; Gait Speed: 4-Meter Walk Gait Speed Test (NIH)) correlated with canonical variate V.

Given our primary interest in the link between behavioral and brain domains and MP, we present here the canonical loadings of the combined model (PSQI, ASR, GMV vs. MP) averaged across over all 100 repeats (Fig. 3). The motor canonical variate in the HCP-YA sample showed a strong correlation with endurance (2-Minute Walk Test), followed by processing speed (Pattern Comparison Task), and low associations to grip strength and gait speed (4-Meter Walk Test) (Fig. 3e). In the eNKI-RS Young sample, the motor canonical variate was positively associated with dexterity (Grooved Pegboard test), motor (TMT) and processing speed (Mouse Practice Task from PennCNB), and cardiorespiratory fitness (VO2max). Interestingly, grip strength showed a negative loading on the motor variates (Fig. 3f).

This component of higher MP was associated with better sleep quality and mild depressive symptoms, exhibiting a consistent pattern across both samples (Fig. 3a, b). This was also evident in the significant correlations between the PSQI and ASR loadings (r_s_ = 0.83, p < 0.001) across the samples. Better sleep quality, particularly self-reported sleep duration and sleep latency/efficiency, indicated relevant loadings in both cohorts. Additionally, the canonical variate was associated with less sleep disturbances, and mild depressive symptoms related to feelings of guilt and self-deprecation (i.e., trouble making decisions) in the HCP-YA sample and lack of energy in the eNKI-RS Young sample. Of note, these associations are based on the canonical loadings, which reflect the associations of the canonical variates and input variables. They do not indicate direct associations between the original variables.

On the brain structural level, we found that various brain regions were associated with MP (Fig. 3c,d). The GMV loadings between the two young samples significantly correlated (r_s_ = 0.39, p < 0.001) with each other. Consistent GMV loadings between both samples were observed in the bilateral thalamus, fusiform gyrus, and parts of the cerebellum. The HCP-YA exhibited loadings in the precentral gyrus. The eNKI-RS Young sample revealed generally higher GMV loadings in a wide range of regions, including the orbitofrontal cortex, precuneus, cingulate cortex, and across the temporal lobes. Cross-loadings, the correlations between sleep quality, depressive symptoms, and GMV variables and the MP variate and between MP measures and the PSQI, ASR, GMV variate, reflected patterns similar to the canonical loadings (Supplementary Figure S4).

### Canonical loadings in mid-to-older adults

**Fig. 4:**
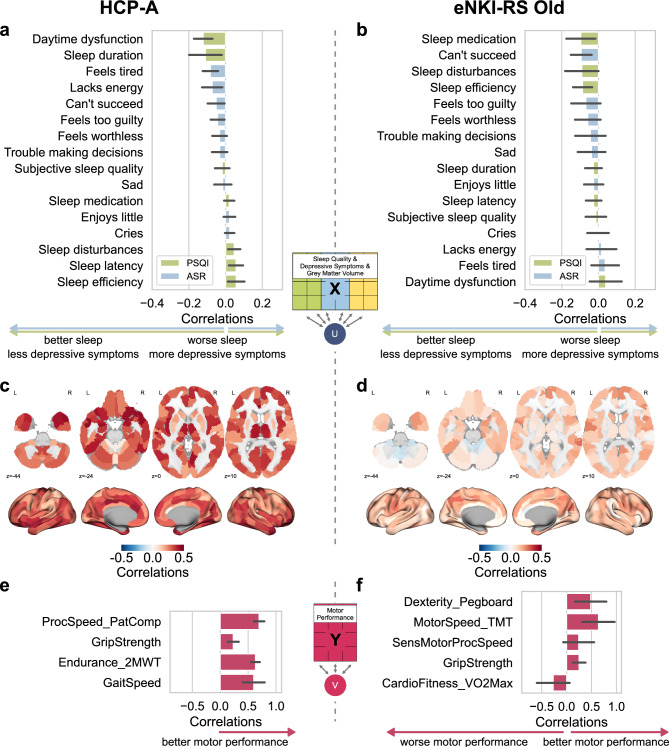
Loadings of regularized Canonical Correlation Analysis of the model combining PSQI, ASR, and GMV vs. motor performance in two samples of mid-to-older adults. a, b, c, d, All variables of sleep quality (PSQI – Pittsburgh Sleep Quality Index), depressive symptoms (ASR – Adult Self Report), and grey matter volume parcels are correlated with the canonical variate U; Negative loadings of PSQI and ASR indicate better sleep quality, and less depressive symptoms, while positive loadings indicate worse sleep quality and more depressive symptoms; e, f, All variables of motor performance (Pegboard: Grooved Pegboard (eNKI-RS); PatComp: Pattern Comparison task from NIH toolbox (NIH); SensMotorProcSpeed: Sensorimotor processing speed – Mouse Practice Task from Penn Computerized Neurobehavioral Battery; TMT: Trail Making task (from Delis-Kaplan Executive Function System); 2MWT: 2-Minute Walk Test (NIH); VO2Max: Cardiovascular fitness estimated from bike test; Gait Speed: 4-Meter Walk Gait Speed Test (NIH)) correlated with canonical variate V. Loadings in the eNKI-RS Old sample are shown solely for the sake of completeness. However, they should not be interpreted due to unstable canonical correlation.

In mid-to-older adults, the combined model (PSQI, ASR, GMV vs. MP) showed small positive canonical loadings for sleep quality and depressive symptoms, while grey matter volume showed higher loadings on the variate associated with the MP variate. This association was observed in the HCP-A sample, but not in the eNKI-RS Old sample, which failed to show robust associations (Fig. 2d and Fig. 4). The motor canonical variate in mid-to-older adults was comprised of processing speed (Pattern Comparison Task), endurance (2-Minute Walk Test), and gait speed (4-Meter Walk Test). The canonical variate of PSQI, ASR, and GMV was associated with less daytime dysfunction and showed minimal loadings of self-reported depressive symptoms, specifically lack of energy and feeling tired (Fig. 4a). Positive loadings of GMV on the canonical variate were observed throughout the brain, suggesting a widespread association of GMV with MP in mid-to-older adults. Brain regions showing the highest loadings include the prefrontal cortex, bilateral amygdala, areas of the orbitofrontal cortex, and thalamic subregions (Fig. 4c).

The cross-loadings of the HCP-A sample showed comparable associations of better sleep quality, less depressive symptoms, and widespread association of GMV with the MP variate (see Supplementary Figure S5). To further assess the robustness of these findings, a sensitivity analysis was conducted, including all available MP measures in the two HCP samples. This analysis showed a similar pattern of associations (see supplementary Figure S6).

### Canonical loadings of behavior and brain rCCA

To further disentangle the motor canonical variates associated with behavioral domains or GMV, we analyzed the loadings from the respective rCCA models. The HCP-YA, HCP-A, and eNKI-RS Young samples demonstrated robust canonical correlations (Fig. 2a-c), while no robust associations were found in the eNKI-RS Old sample, probably because of the low sample size in this group (Fig. 2d). Therefore, the loadings are reported for completeness but not discussed further. Statistical comparisons of the loadings between samples showed significant correlations for the rCCA models of GMV vs. MP: HCP-YA and eNKI-RS Young (r_s_ = 0.34, p < 0.001), HCP-YA and HCP-A (r_s_ = 0.34, p < 0.001), and eNKI-RS Young and HCP-A (r_s_ = 0.56, p < 0.001). Conversely, the loadings from the behavioral rCCA (PSQI, ASR vs. MP) models showed no significant correlations between the samples.

Regarding the MP domains, endurance, grip strength, motor speed, and dexterity showed varying degrees of association with the motor canonical variate across samples, with higher grip strength linked to fewer somatic symptoms and longer sleep in HCP-A but more somatic symptoms in eNKI-RS Young. In contrast, processing speed, cardiorespiratory fitness, and gait speed emerged as stronger correlates of the motor variate in the brain rCCA models (Supplementary Figure S7 & S8).

## Discussion

We found robust and replicable multivariate associations between sleep quality, depressive symptoms, and GMV with MP, particularly in two samples of young adults. Across all samples, higher MP was reflected in a motor canonical variate that was positively associated with a variate characterized by better sleep quality. Depressive symptoms showed a differential pattern: In young adults, mild, sub-clinical depressive symptoms (feelings of guilt, self-deprecation, lack of energy, and fatigue) positively loaded on the variate, which was associated with the motor canonical variate reflecting higher MP, whereas, in mid-to-older adults, the variate of fewer depressive symptoms was associated with the variate of higher MP. GMV showed the most robust and consistent canonical correlation with MP across all age groups and individual domains. The combination of all domains into a single rCCA model strengthened the associations. However, rCCA models with sleep quality combined with depressive symptoms or just GMV showed similarly high associations in the HCP-YA, eNKI-RS Young, and HCP-A samples, respectively. This may indicate that behavioral and brain structural factors are linked through partly distinct associations with MP, although our analyses did not formally differentiate shared versus unique variance between domains. Future studies employing variance partitioning or mediation approaches are necessary to elucidate the independence or overlap between these modes of association. Shortcomings of this study include the relatively small sample size for brain-behavior associations^67,68^, but we did not find other samples that contain similar behavioral data. Moreover, the MP tasks available within each cohort only allowed for conceptual and qualitative replication^66^. While we restricted our analyses to the same MP measures within the HCP samples and within the eNKI-RS samples, task differences remained between the HCP and the eNKI-RS. These differences may have contributed to the observed inconsistencies across samples. To formally test construct equivalence across MP measurements, future studies could employ complementary approaches, such as structural equation modeling (SEM). In addition, future studies should consider the role of further psychological factors, such as anxiety, stress, or cognitive limitations. Furthermore, physical factors (e.g., cardiovascular and metabolic health) and medication use may influence MP, particularly in older adults. To control for the potential confounding effect of sex, we regressed out the effect in all rCCA models. However, sex imbalances in specific samples, such as eNKI-RS Old, may have potentially introduced some bias. The failure to replicate associations among mid-to-older adults in the eNKI-RS Old sample may be explained by a combination of small sample size, increased variance in motor performance with aging, and a more heterogeneous participant group. Future studies should also investigate the generalizability of the findings, particularly in mid-to-older adults. In addition, directionality/causality cannot be inferred based on rCCA method and in the context of the cross-sectional design.

### Differential brain and behavior association with MP

In the HCP-YA sample, combining behavioral factors and GMV showed slightly higher canonical correlations with MP compared to the model based on GMV-only, but not compared to the model based on both behavioral factors. However, these differences were not statistically significant and should therefore be interpreted with caution. In the eNKI-RS Young and HCP-A samples, canonical correlations were nearly identical between the combined and GMV-only model. Therefore, the associations of behavior and GMV with MP appear to vary across samples and may be driven by either behavioral factors or brain structure, rather than their joint contribution. A possible explanation for these findings is the lack of a common link between brain structure and sleep quality/depressive symptoms, as highlighted previously^8,34,69^. Similarly, a study examining the predictability of global cognition by brain and demographic information found that demographic information alone was more effective at predicting cognition than brain features. Furthermore, the models that combined brain and demographic information showed similar or even worse results^70^. In our study, this is consistent with the results of the HCP-YA sample, where the behavioral rCCA model showed the strongest associations with MP, and the addition of GMV did not increase the correlation. In contrast, in the eNKI-RS Young and HCP-A samples, GMV-only rCCA models demonstrated higher hold-out associations and redundancy indices, possibly due to the influence of other motor tasks or age-related differences.

We found that a motor canonical variate characterized by endurance, grip strength, and dexterity was associated with behavioral factors. As the motor variate correlated weakly with the more cognitive task (processing speed), it may represent a more fundamental aspect of motor function. Endurance showed the highest loadings in both HCP cohorts, whereas cardiovascular fitness in the eNKI-RS Young sample showed no loading, possibly due to differences in measurement methods. Moreover, short sleep duration was associated with worse MP, which aligns with the literature linking sleep deprivation and poor sleep quality with lower endurance^17^. Cardiovascular endurance was further linked to sleep latency in a cohort of middle-aged and older adults, which we similarly observed in the HCP-YA sample^71^. Grip strength showed high loadings in the eNKI-RS Young and HCP-A samples, positively associated with fewer depressive symptoms, which aligns with a previous study^20^. Interestingly, a recent study found that short sleep duration increased the risk of developing depressive symptoms, but this effect was attenuated in participants with high grip strength^72^. In the HCP-A sample, we observed lower depressive symptoms being associated with higher endurance and grip strength, consistent with previous literature^20,21^. Measures of processing speed seemed less relevant, contrary to the findings of a study of a mixed cohort of depressed and non-depressed people, which found a positive association between the pegboard task, processing speed, and depressive symptom severity but not the diagnosis of depression^26^. The results from our multivariate approach showed that a combination of normal sleep duration and efficiency, together with less depressive symptoms in mid-to-older adults and more depressive symptoms in younger adults, is associated with higher MP.

The association with GMV showed a motor canonical variate driven more by cognitive aspects of processing speed, dexterity, gait and endurance (Supplementary Figure S8). Previous studies have identified associations between MP and GMV in various brain regions. Macroanatomical brain correlates with manual MP were found for cerebral GMV, but not consistently for cerebellar GMV^29,73^. A recent study found associations between GMV atrophy and manual dexterity in more fine-grained parietal areas and gross motor function in the temporal regions^74^. Fitness and physical activity have been primarily associated with GMV differences in the prefrontal cortex and the hippocampus^75^. In this study, we found high loadings in regions that have previously been linked to motor control and sleep and mood regulation. The thalamus plays a central role in motor relay, sleep–wake regulation and structural alterations have been linked to depressive disorder^76–78^. While the cerebellum is traditionally associated with motor coordination, recent studies have observed structural differences between MDD patients and healthy controls^79,80^. However, its potential role in sleep disturbances remains to be investigated. In mid-to-older adults, we observed loadings in the amygdala, an important region in emotional regulation. This region has been identified in a recent large-scale meta-analysis of sleep disorders and its role in depression has received a lot of attention^81,82^. The orbitofrontal cortex, which demonstrated loadings in the eNKI-RS Young and the HCP-A samples, has been associated with depressive symptomatology and insomnia^83^.

### Different associations with MP in younger and mid-to-older adults

The relationship between the behavioral and brain domains with MP exhibited distinct patterns between younger and mid-to-older adults (Fig. 3 and Fig. 4). In younger adults, a variate of better sleep characteristics, such as increased duration, reduced latency, higher sleep efficiency, and mild depressive symptoms, was associated with higher MP. We speculate that the canonical correlation between the variate characterized by more mild, sub-clinical depressive symptoms and the variate reflecting higher MP in young participants might be self-doubt or personal sensitivity to conduct their task or answer the questions as potential motivational factor^84^, higher neuroticism/anxiety due to perfectionism-related hyperarousal^85^, or increased exercise as a compensatory strategy in those with generally higher depressive symptoms^86^, given that the participants were from the general population and did not meet diagnostic criteria for clinical depression. The very low severity scores of depressive symptoms in our young adult samples further explain such a (surprisingly) small correlation between the variates of more depressive symptoms and higher MP, while mid-to-older adults in the HCP-A have more depressive symptoms variability and, therefore, a variate of better sleep quality, and less somatic-related depressive symptoms was correlated with the variate reflecting better MP.

Ageing affects MP through multiple aspects, such as sarcopenia, mobility issues, cardiovascular and metabolic health, and cognitive decline^87–89^. Ageing differently impacts sleep in mid-to-older adults, the sleep becomes more fragmented, sleep latency increases, and slow-wave sleep decreases^42^. In addition, the nature of depressive symptoms shifts in older people, with an increased prevalence of somatic complaints, whereas feelings of guilt may be more prevalent in younger adults^43,90^. These changes occur with broader biological changes associated with aging. These processes include a reduction in the dopaminergic signaling, which may contribute to psychomotor slowing, slowed processing speed, and impaired motivation^91^. Similarly, low-grade/chronic age-related inflammation can disrupt sleep regulation or interact with dopaminergic pathways, thereby heightening the risk for depressive symptoms and reduced motor function^91–94^. Furthermore, changes in white matter structure have been linked to reductions in processing speed and changes in sleep architecture^42,95^. These age-related differences underscore the intricate interplay between biological and behavioral factors that influence MP throughout the lifespan.

## Conclusions

Our findings revealed a complex pattern of associations between behavioral and brain structural factors with MP. Using a machine learning framework to ensure the robustness of our results, we found that canonical variates of better sleep quality and mild depressive symptoms were associated with canonical variates of better MP in young adults. This was conceptually replicated in a second young cohort. In a cohort of mid-to-older adults, we observed that variates of healthy sleep and fewer depressive symptoms were associated with variates of better MP. Brain structure was associated with more cognitive-driven MP. We hope that these findings increase the incentive regarding the importance of sleep quality and depressive symptoms to improve motor functioning of ordinary individuals in society, professional athletes, patients with motor-related neurodegenerative and psychiatric conditions, including Parkinson’s disease or MDD. To extend these findings and assess the reproducibility of our findings, future large-scale studies using open data sharing and international consortia (e.g., ENIGMA-Sleep^96^) should assess a range of motor behaviors together with a broader range of phenotypic measures, such as personality, environmental, and lifestyle factors in younger and mid-to-older adults, which are critical aspects toward personalized medicine. Longitudinal assessments would help in elucidating the causal relationships between behavioral and brain structural aspects of MP.

## Supplementary Information

Supplementary Information.

## Data Availability

We used publicly available datasets. Access to the Human Connectome Project can be requested after registering and accepting the data-use terms (db.humanconnectome.org). Access to the HCP-A can be requested and accessed through the NIMH Data Archive, further information can be found on website of the HCP (https://www.humanconnectome.org/study/hcp-lifespan-aging/data-releases). Data of the enhanced Nathan Kline Institute Rockland Sample can be accessed as outlined here: https://fcon_1000.projects.nitrc.org/indi/enhanced/access.html. The code used for the rCCA and machine learning framework computation, including examples on how to conduct CCA analyses is available at https://github.com/anaston/cca_pls_toolkit. See also the accompanying tutorial paper for in-depth explanations of the framework and analysis^51^. CAT12 can be downloaded at https://neuro-jena.github.io/cat/index.html#DOWNLOAD.
