# Supplementary material for: Multivariate associations of motor performance, sleep quality, depressive symptoms, and grey matter volume in younger and mid-to-older adults
Source: Sci Rep. 2026 Jan 10;16:1318. doi: 10.1038/s41598-025-34951-y (PMC12796161; doi:10.1038/s41598-025-34951-y)
Supplement: Supplementary file 1 — Supplementary Information. [file 41598_2025_34951_MOESM1_ESM.pdf]

Supplementary material:

Multivariate associations of motor performance, sleep quality, depressive symptoms, and grey matter volume in younger and mid-to-older adults

Vincent Küppers et al.

Table of Contents

Methods **2**

Participants and phenotypic data **2**

HCP-YA 2

eNKI-RS Young 2

HCP-A 3

eNKI-RS Old 3

Pittsburgh Sleep Quality Index and Adult Self-Report DSM-oriented scales 3

Results **4**

References **17**

Methods

Participants and phenotypic data

Data from three different publicly available cohorts was analysed: the Human Connectome Project Young Adult (HCP-YA, S1200 release), the Human Connectome Project Aging (HCP-A, 2.0 release), and the enhanced Nathan Kline Institute-Rockland Sample (eNKI-RS) [1–3]. To investigate potential age-related differences, participants were divided into two groups: younger adults (HCP-YA: 22-37 years; eNKI-RS Young: 18-40 years) and mid-to-older adults (HCP-A: 50-85 years; eNKI-RS Old: 50-85 years). After excluding participants with missing data and low-quality neuroimaging data, a total of 1954 participants (1114 women) were included.

HCP-YA

1086 participants (587 female, 22-37 years). Sleep quality was assessed using seven components computed in the Pittsburgh Sleep Quality Index (PSQI) [4]. Depressive symptoms were measured using the relevant items of the Adult Self-Report (ASR) for ages 18-59 associated with depressive Disorder [5,6]. Items not included in the ASR version for older adults for ages 60+ (OASR) and sleep-related questions were excluded. To avoid items with a variance close to zero, which could distort the covariance structure and reduce the interpretability and stability of the rCCA analysis, items where more than 95% of the participants answered with the same response were excluded. Motor-related phenotypes were acquired using the NIH Toolbox normalised scores for (1) Grip strength (Strength), an assessment of upper body strength using a dynamometer, (2) 2-Minute Walk Test (Endurance), a test of physical fitness, cardiovascular endurance by measuring the distance covered by a participant in 2 minutes time, (3) 4-Meter Walk Gait Speed Test (Gait speed), a test of functional mobility where participants are asked to walk at their usual pace for four meters [7], and (4) Pattern Completion Processing Speed (Processing Speed), an assessment of the speed of processing by asking participants to identify as quickly as possible if two pictures are the same or not [8]. The 9-Hole Pegboard Test (Dexterity) is an assessment of fine motor dexterity by measuring the time that it takes participants to place pegs in 9 holes and remove them [7]. This task was only included in the supplementary analysis (Figure S6) to ensure the comparability of motor tasks with HCP-A, for which this task was not assessed. For the ASR and PSQI, higher scores indicate higher symptom severity, i.e., more depressive symptoms and worse sleep quality. For all motor measures, higher scores indicate better motor performance, i.e., stronger, faster.

eNKI-RS Young

230 participants (128 female, 18-40 years). Sleep quality and depressive symptoms were measured as in HCP-YA. To make the MP measures comparable to the human connectome project, we took the (1) grip strength of the dominant hand (Strength), (2) VO2max by bike test (Endurance), a more direct measurement of cardiorespiratory fitness by measuring the heart rate on a stationary bike (3) Grooved Pegboard Test (Dexterity), similar to the 9-hole pegboard but the participants are required to place 25 pegs, which are uniquely shaped (4) Sensorimotor processing speed - Mouse Practice task (Processing Speed), a test in which participants have to react as quickly as possible to green squares appearing on a computer screen by moving the cursor and clicking on them (5) Trail Making Test Motor speed of the Delis-Kaplan Executive Function System toolbox (TMT Motor Speed), a condition of the TMT where the participants are asked to draw a line over a dotted line as quickly as possible [7,9–11]. It should be noted that the VO2max calculated by the bike test can also be considered an indirect measure of

cardiorespiratory fitness. The submaximal VO2max and finger tapping tasks were both excluded due to the relatively small number of participants who performed these measures.

HCP-A

358 participants (200 female, 50-85 years). Sleep quality was assessed using the PSQI questionnaire, and depressive symptoms were evaluated using the OASR, as described above. MP measures are based on the NIH Toolbox normalised scores for Grip strength (Strength), 2-Minute Walk Test (Endurance), 4-Meter Walk Gait Speed Test (Gait speed), and Pattern Completion Processing Speed (Processing Speed). The raw score of Trail Making Test A [7,8,12] was included in the sensitivity analysis (Figure S6).

eNKI-RS Old

280 participants (199 female, 50-85 years). Sleep quality and depressive symptoms were measured as in HCP-A. Motor-related phenotypes were identical to those of the eNKI-RS Young sample.

Pittsburgh Sleep Quality Index and Adult Self-Report DSM-oriented scales

The Pittsburgh Sleep Quality Index (PSQI) is a widely utilised self-report questionnaire created to evaluate sleep quality across seven components. Higher scores on these components and scales indicate poorer sleep quality. The test demonstrates high internal consistency (Cronbach's alpha = 0.83) and strong test-retest reliability (r = 0.85) [4]. The ASR DSM-oriented scales, including the scale for depressive symptoms, were derived from ratings by 21 experts. The ASR questionnaire items were assigned to problem scales consistent with DSM-IV diagnostic criteria. Testing these scales in a US representative sample demonstrated strong internal consistency and high test-retest reliability. Statistically derived syndromes from the same sample exhibited moderate to strong correlations with the DSM-oriented scales [13].

MRI data acquisition

T1-weighted scans were acquired using the MPRAGE sequence in all three cohorts. In the HCP-YA, this was done using a single Siemens 3T Skyra scanner with a resolution of 0.7mm isotropic voxels [3]. In the HCP-A cohort, MRI data was acquired across four sites using Siemens Prisma 3T MRI scanners with a resolution of 0.8mm isotropic voxels [1]. In the eNKI-RS cohort, data was acquired using Siemens 3T Tim Trio scanner with a resolution of 1mm isotropic voxels [2].

Results

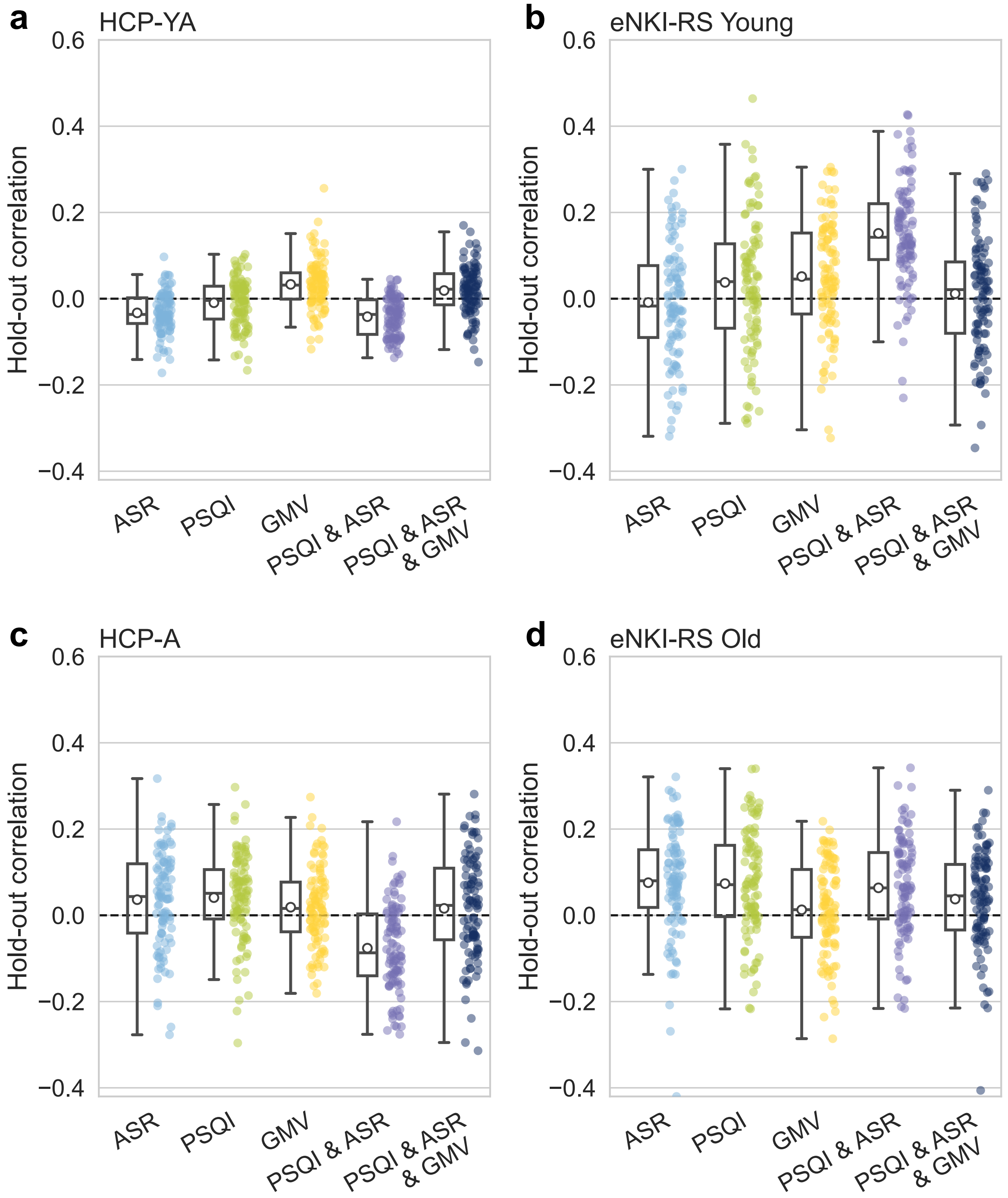

Figure S1. Second mode: Individual and combined regularized Canonical Correlation Analysis (rCCA) results across four samples. Multivariate associations between depressive symptoms (ASR), sleep quality (PSQI), grey matter volume (GMV), and their combinations with measures of motor performance. Canonical correlation values correspond to the hold-out canonical correlations, computed in 100 outer splits for every model, and in each sample.

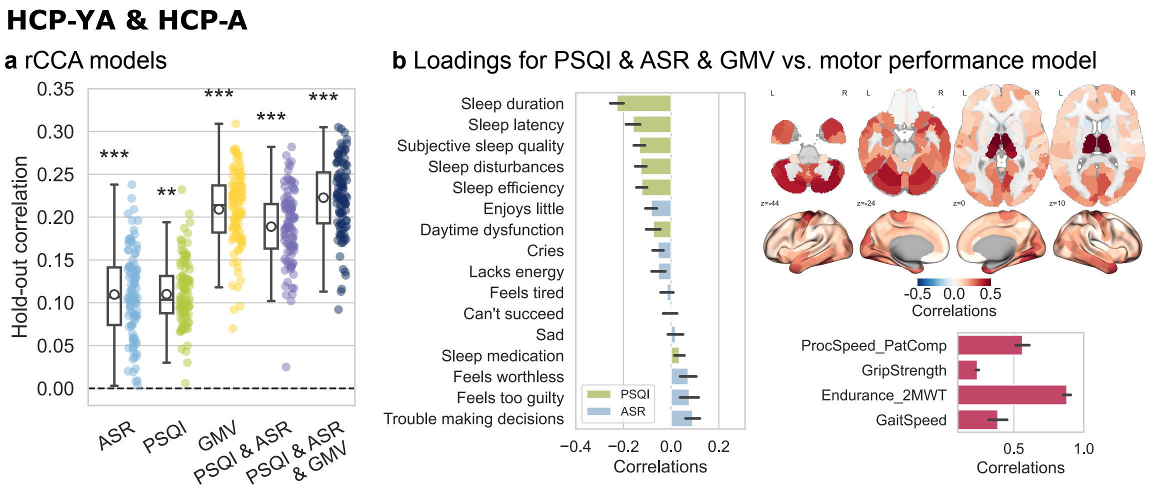

Figure S2. Combined sample of HCP-YA and HCP-A cohort (n = 1631, 894 female, age range = 22-85). **a**, Individual and combined regularized Canonical Correlation Analysis (rCCA) 100 times repeated hold-out correlations. **b**, Canonical loadings of rCCA of the model combining PSQI & ASR & GMV vs. motor performance. All variables of sleep quality (PSQI – Pittsburgh Sleep Quality Index), depressive symptoms (ASR – Adult Self Report), grey-matter-volume parcels are correlated with the canonical variate U, negative loadings of PSQI and ASR indicate better sleep quality, and less depressive symptoms, while positive loadings indicate worse sleep quality and more depressive symptoms; and all variables of motor performance (PatComp: Pattern Comparison task from NIH toolbox; Grip Strength; 2MWT: Two-Minute Walk Test; Gait Speed: 4-Meter Walk Gait Speed Test (NIH)) correlated with canonical variate V.

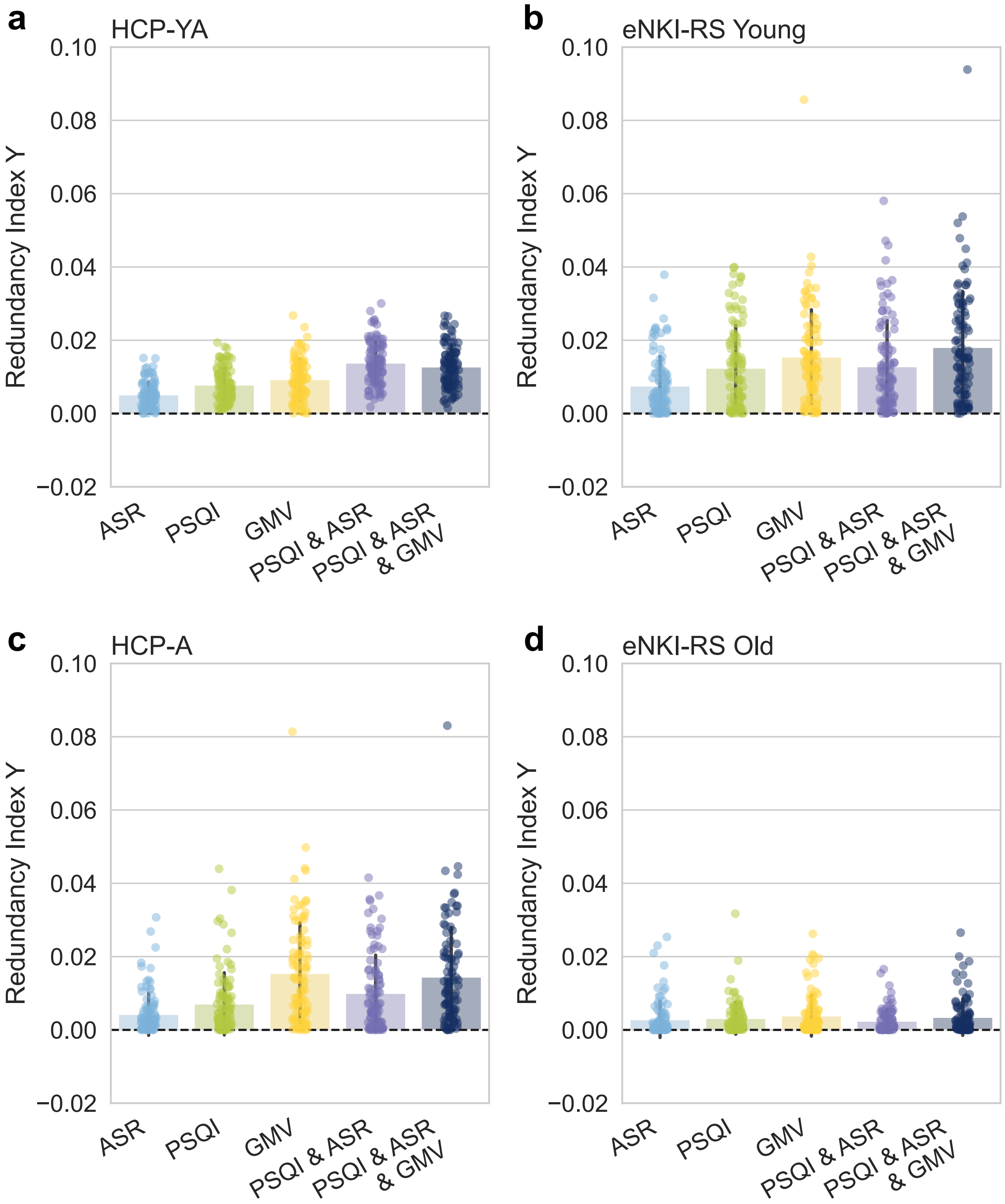

Figure S3. Redundancy Index of Y from the first mode. Model performance of rCCA models including depressive symptoms (ASR), sleep quality (PSQI), grey matter volume (GMV), and their combinations with measures of motor performance (MP). Redundancy index of Y represents the proportion of variance in the Y variables (MP) that can be explained by the canonical variate derived from X. Redundancy indices were estimated across 100 outer splits for each model, and in each sample.

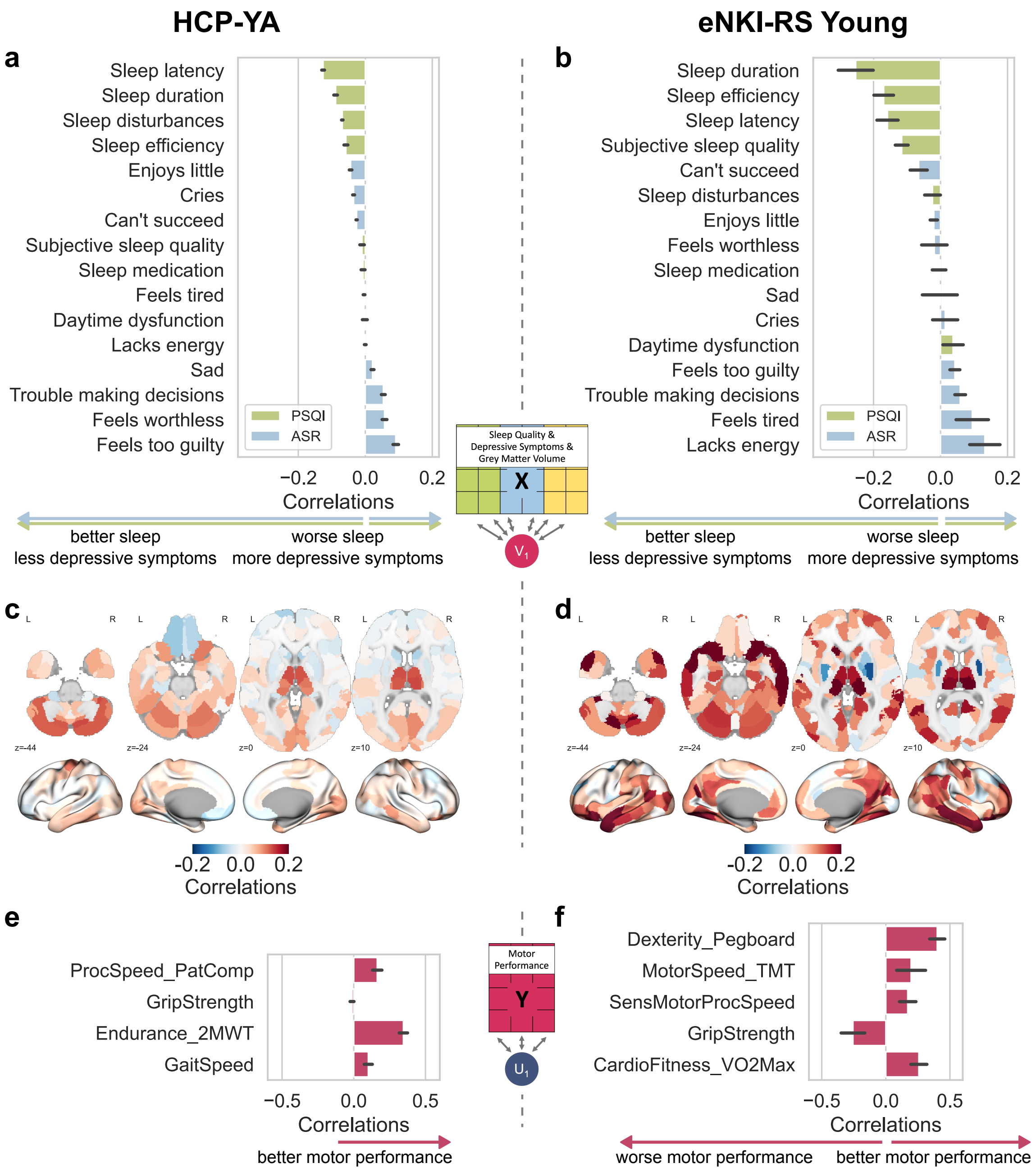

Figure S4. Cross-loadings of regularized Canonical Correlation Analysis of the model combining PSQI, ASR, and GMV vs. motor performance in younger adults. a, b, c, d, All variables of sleep quality (PSQI – Pittsburgh Sleep Quality Index), depressive symptoms (ASR – Adult Self Report), and grey matter volume parcels are correlated with the canonical variate V (from MP); Negative cross-loadings of PSQI and ASR indicate better sleep quality, and less depressive symptoms, while positive cross-loadings indicate worse sleep quality and more depressive symptoms; e, f, All variables of motor performance (Pegboard: Grooved Pegboard (eNKI-RS); PatComp: Pattern comparison task from NIH toolbox (NIH); SensMotorProcSpeed: Sensorimotor processing speed – Mouse Practice Task from Penn Computerized Neurobehavioral Battery; TMT: Trail Making task (from Delis-Kaplan Executive Function System); 2MWT: 2-Minute Walk Test (NIH); VO2Max: Cardiovascular fitness estimated from bike test; Gait Speed: 4-Meter Walk Gait Speed Test (NIH)) correlated with canonical variate U (from PSQI, ASR, and GMV).

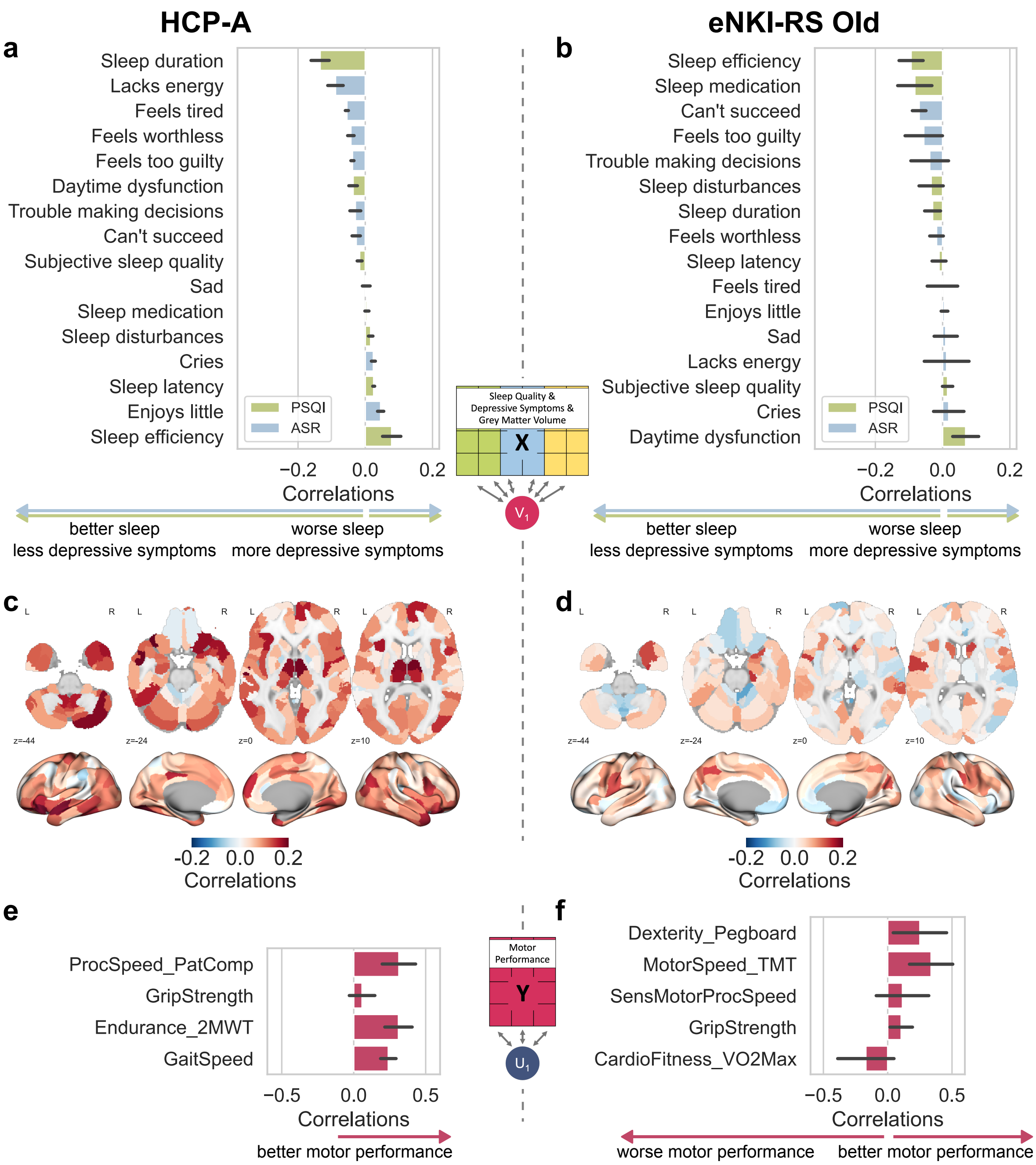

Figure S5. Cross-loadings of regularized Canonical Correlation Analysis of the model combining PSQI, ASR, and GMV vs. motor performance in mid-to-older adults. a, b, c, d, All variables of sleep quality (PSQI – Pittsburgh Sleep Quality Index), depressive symptoms (ASR – Adult Self Report), and grey matter volume parcels are correlated with the canonical variate V (from MP); Negative cross-loadings of PSQI and ASR indicate better sleep quality, and less depressive symptoms, while positive cross-loadings indicate worse sleep quality and more depressive symptoms; e, f, All variables of motor performance (Pegboard: Grooved Pegboard (eNKI-RS); PatComp: Pattern comparison task from NIH toolbox (NIH); SensMotorProcSpeed: Sensorimotor processing speed – Mouse Practice Task from Penn Computerized Neurobehavioral Battery; TMT: Trail Making task (from Delis-Kaplan Executive Function System); 2MWT: 2-Minute Walk Test (NIH); VO2Max: Cardiovascular fitness estimated from bike test; Gait Speed: 4-Meter Walk Gait Speed Test (NIH)) correlated

with canonical variate U (from PSQI, ASR, and GMV). Cross-loadings in the eNKI-RS Old sample are shown solely for the sake of completeness. However, they should not be interpreted due to unstable canonical correlation.

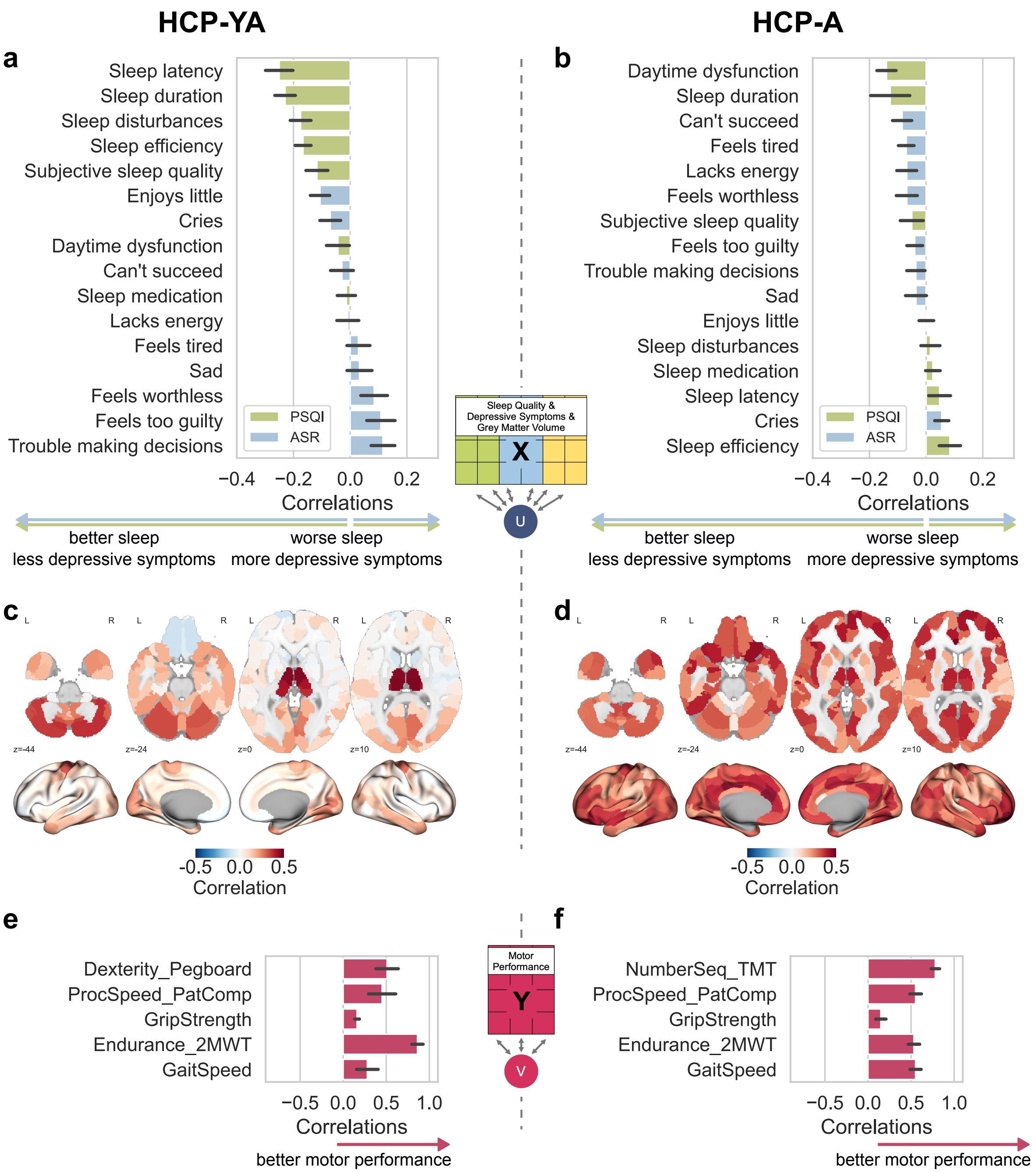

Figure S6. Loadings of regularized Canonical Correlation Analysis of the model combining PSQI, ASR, and GMV vs. motor performance including all available motor measures. a, b, c, d, All variables of sleep quality (PSQI – Pittsburgh Sleep Quality Index), depressive symptoms (ASR – Adult Self Report), and grey matter volume parcels are correlated with the canonical variate U; Negative loadings of PSQI and ASR indicate better sleep quality, and less depressive symptoms, while positive loadings indicate worse sleep quality and more depressive symptoms; e, f, All variables of motor performance correlated with canonical variate V.

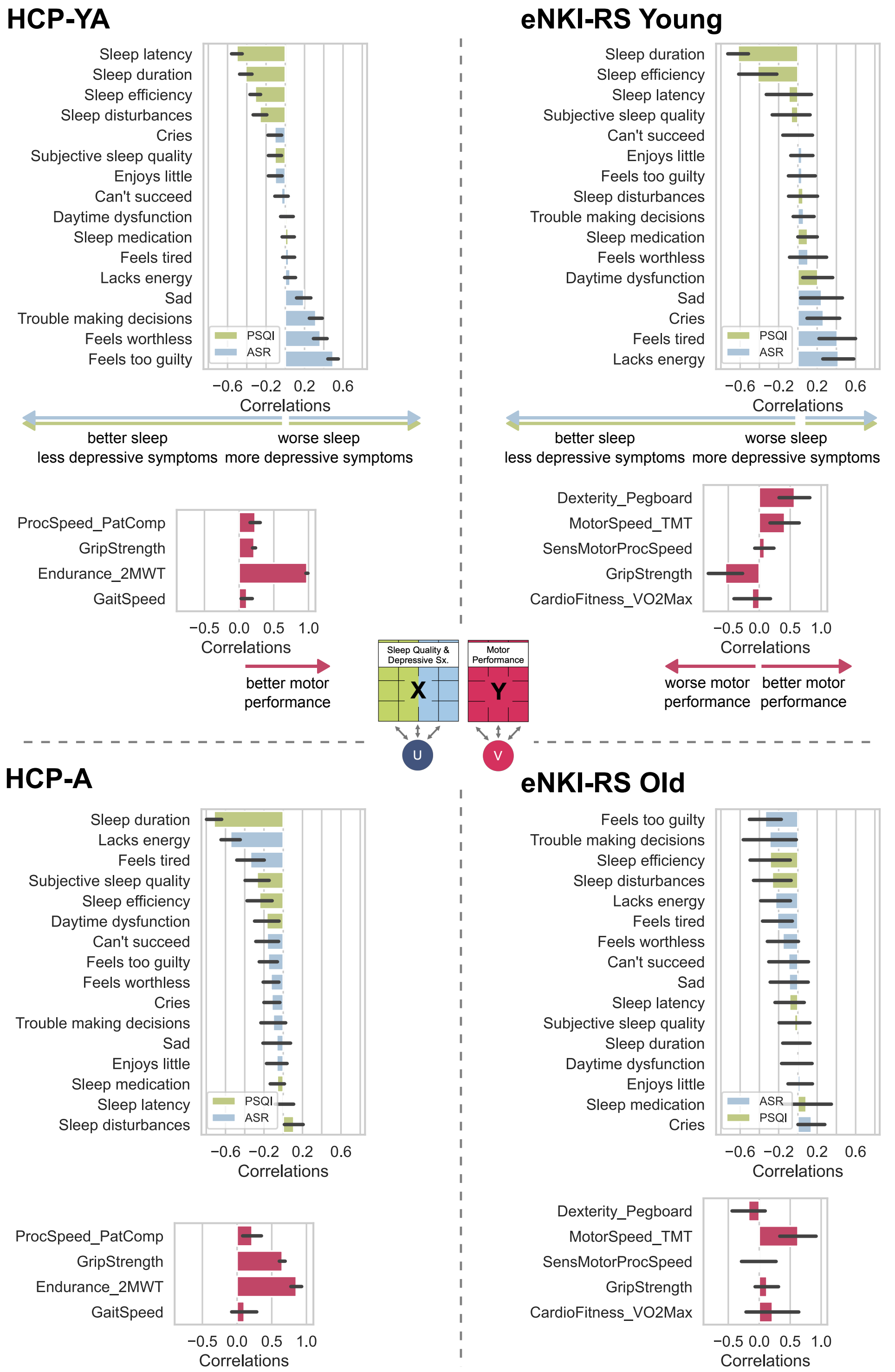

Figure S7. Loadings of regularized Canonical Correlation Analysis of the model combining PSQI & ASR vs. motor performance. All variables of sleep quality (PSQI – Pittsburgh Sleep Quality Index), depressive symptoms (ASR – Adult Self Report) are correlated with the canonical variate U. Negative loadings of PSQI and ASR indicate better sleep quality, and less depressive symptoms, while positive loadings indicate worse sleep quality and more depressive symptoms; all variables of motor performance (PatComp: Pattern Comparison task from NIH toolbox; SensMotorProcSpeed: Sensorimotor processing speed – Mouse Practice Task from Penn Computerized Neurobehavioral Battery; 2MWT: Two-Minute Walk Test; Grip Strength; Grooved Pegboard (eNKI-RS); VO2Max: Cardiovascular fitness estimated from bike test) correlated with canonical variate V.

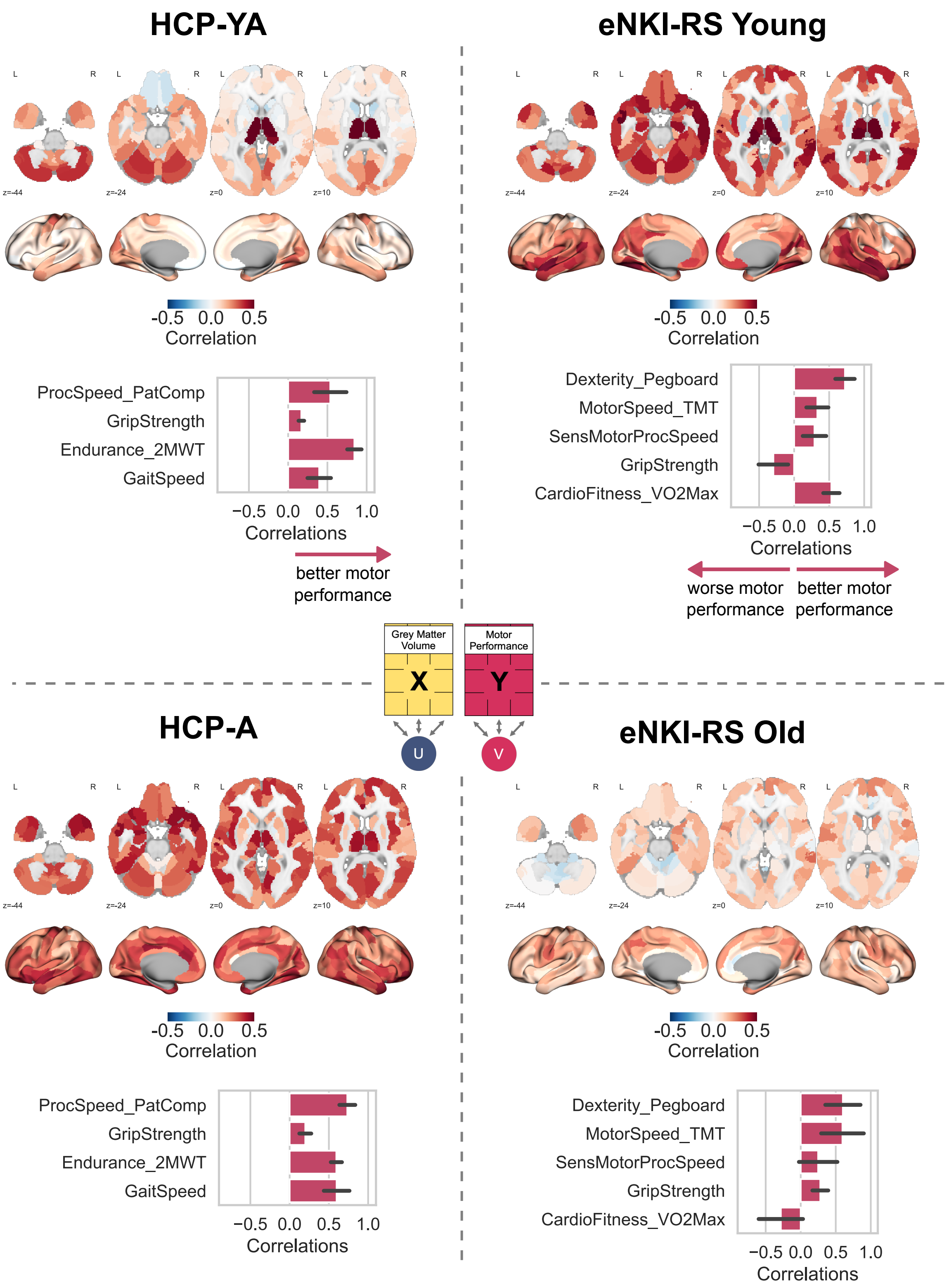

Figure S8. Loadings of regularized Canonical Correlation Analysis of the model GMV vs. motor performance. All variables of grey-matter-volume are correlated with the canonical variate U. Negative loadings of PSQI and ASR indicate better sleep quality, and less depressive symptoms, while positive loadings indicate worse sleep quality and more depressive symptoms; all variables of motor performance (PatComp: Pattern Comparison task from NIH toolbox; SensMotorProcSpeed: Sensorimotor processing speed – Mouse Practice Task from Penn Computerized Neurobehavioral Battery; 2MWT: Two-Minute Walk Test; Grip Strength; Grooved Pegboard (eNKI-RS); VO2Max: Cardiovascular fitness estimated from bike test) correlated with canonical variate V.

A visual comparison of the motor canonical variates of the behavioral rCCAs revealed that the endurance task showed the highest loadings in the HCP-YA (r = 0.98) and HCP-A (r = 0.85) samples (Figure S5). Conversely, in the eNKI-RS Young sample, VO2max showed no association with the motor variate. The pegboard task loaded on the motor variate in eNKI-RS Young sample (0.57). Interestingly, grip strength was associated with the motor canonical variates for both eNKI-RS Young and HCP-A. In the eNKI-RS Young sample, lower grip strength (r = -0.55) was associated with higher depressive symptoms, especially somatic aspects (Lacks energy r = 0.42, Feels tired r = 0.41). For the HCP-A sample, higher grip strength (r = 0.65) was linked to longer sleep duration (r = 0.72) and less somatic symptoms (Lacks energy r = -0.55, Feels tired r = -0.34). Processing speed was not really associated with the motor variate in any of the samples. Comparing the motor variates of the brain rCCA models, processing speed showed higher loadings in all three samples (HCP-YA r = 0.54, eNKI-RS Young r = 0.29, HCP-A r = 0.74) (Figure S6). Gait speed loaded on the motor variate in both the HCP-YA (r = 0.4) and HCP-A (r = 0.6) samples. The pegboard task showed the highest loadings on the motor variate from the eNKI-RS Young sample, with r = 0.73. Further endurance loaded on the motor variate in the HCP-YA (r = 0.84), HCP-A (r = 0.6) and cardiorespiratory fitness in the eNKI-RS Young (r = 0.5).

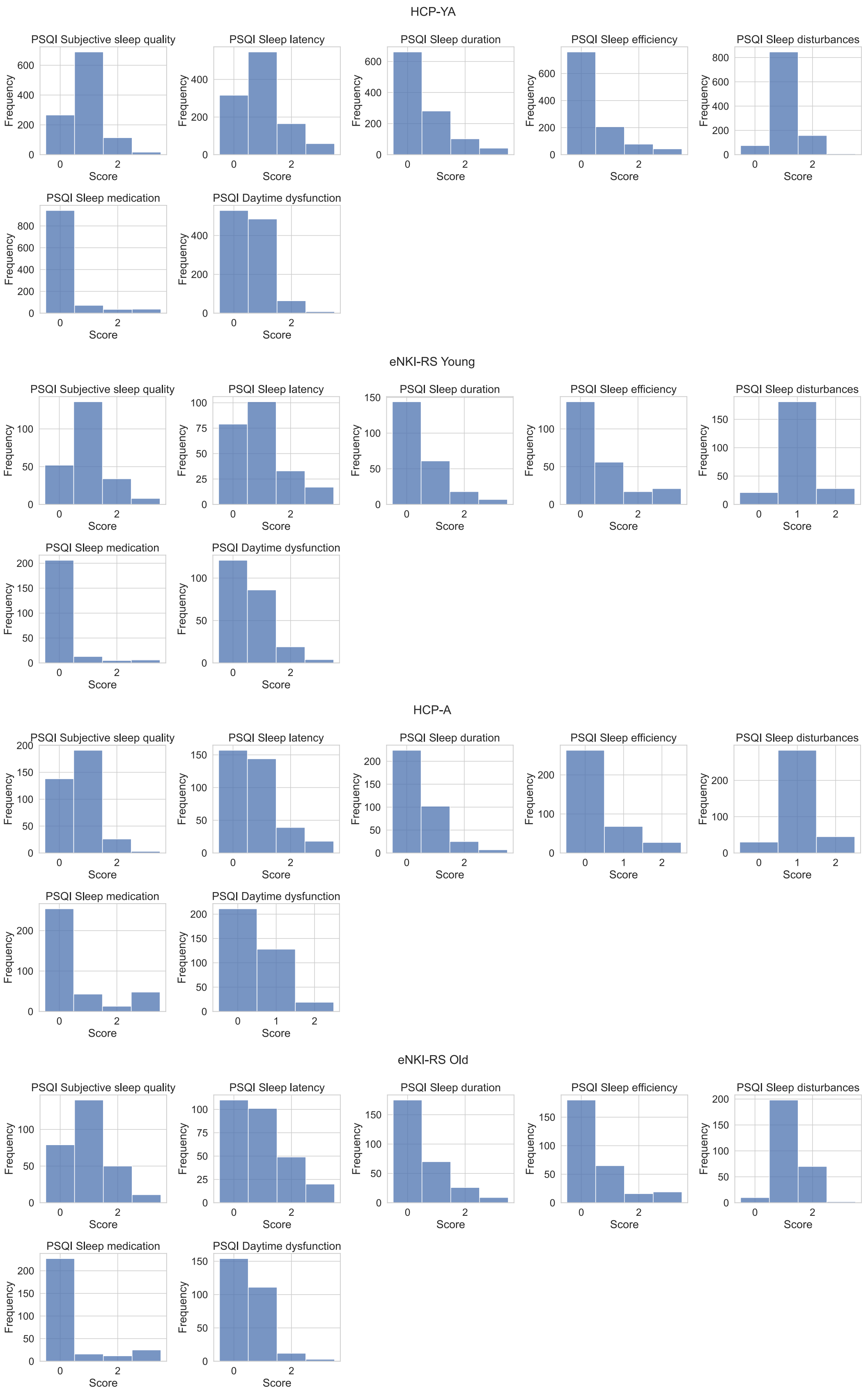

Figure S9. Distribution of the seven Pittsburgh Sleep Quality Index (PSQI) components. Each component score ranges from 0 to 3, with higher scores indicating poorer sleep quality.

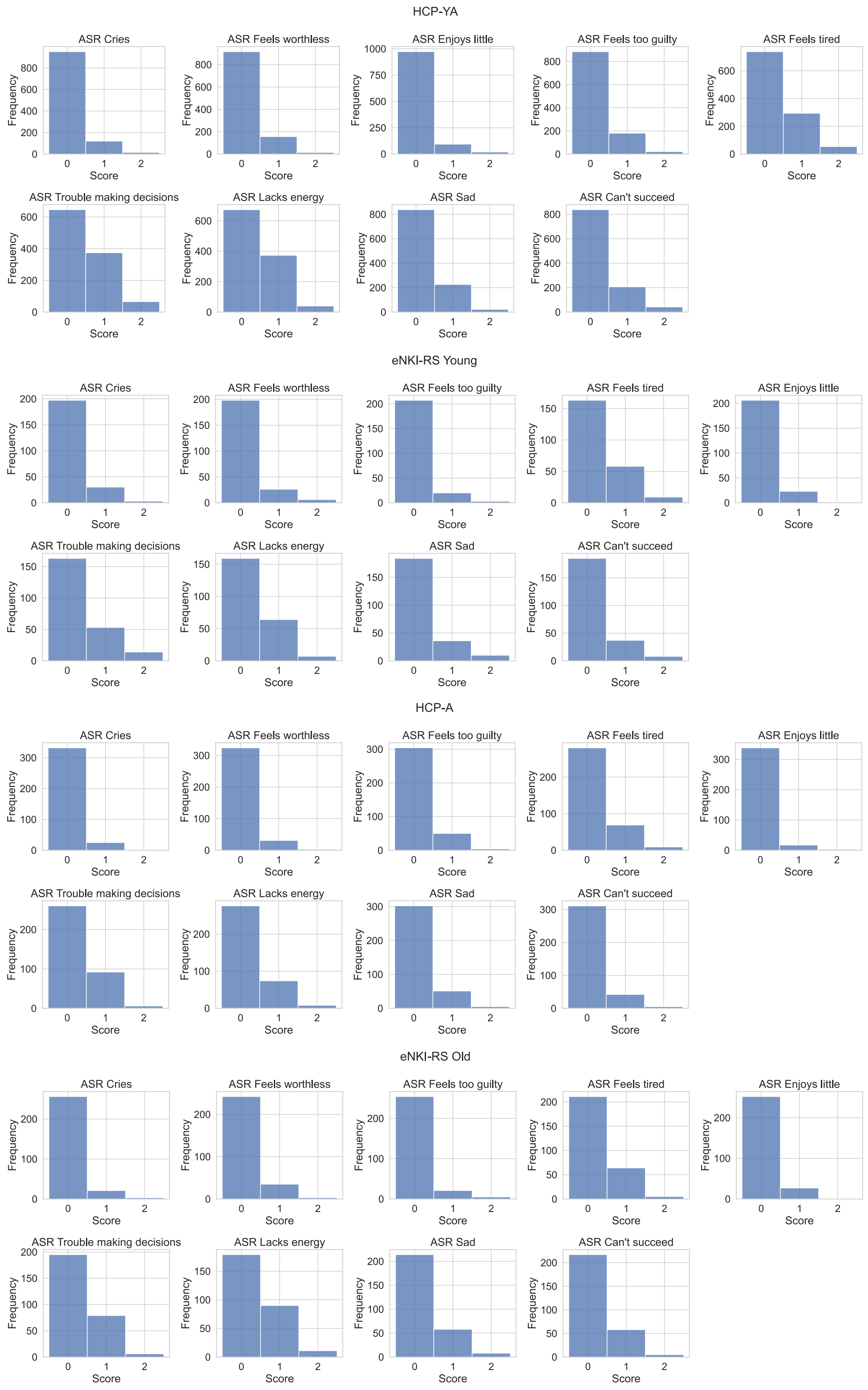

Figure S10. Distribution of the depression-related items from the Adult Self Report (ASR). Each item scores ranges from 0 (Not True) to 2 (Very True or Often True). For each sample, only the items included in the analyses are shown. Some items were excluded based on variance close to zero (more than 95 % of the participants gave the same response within the sample).

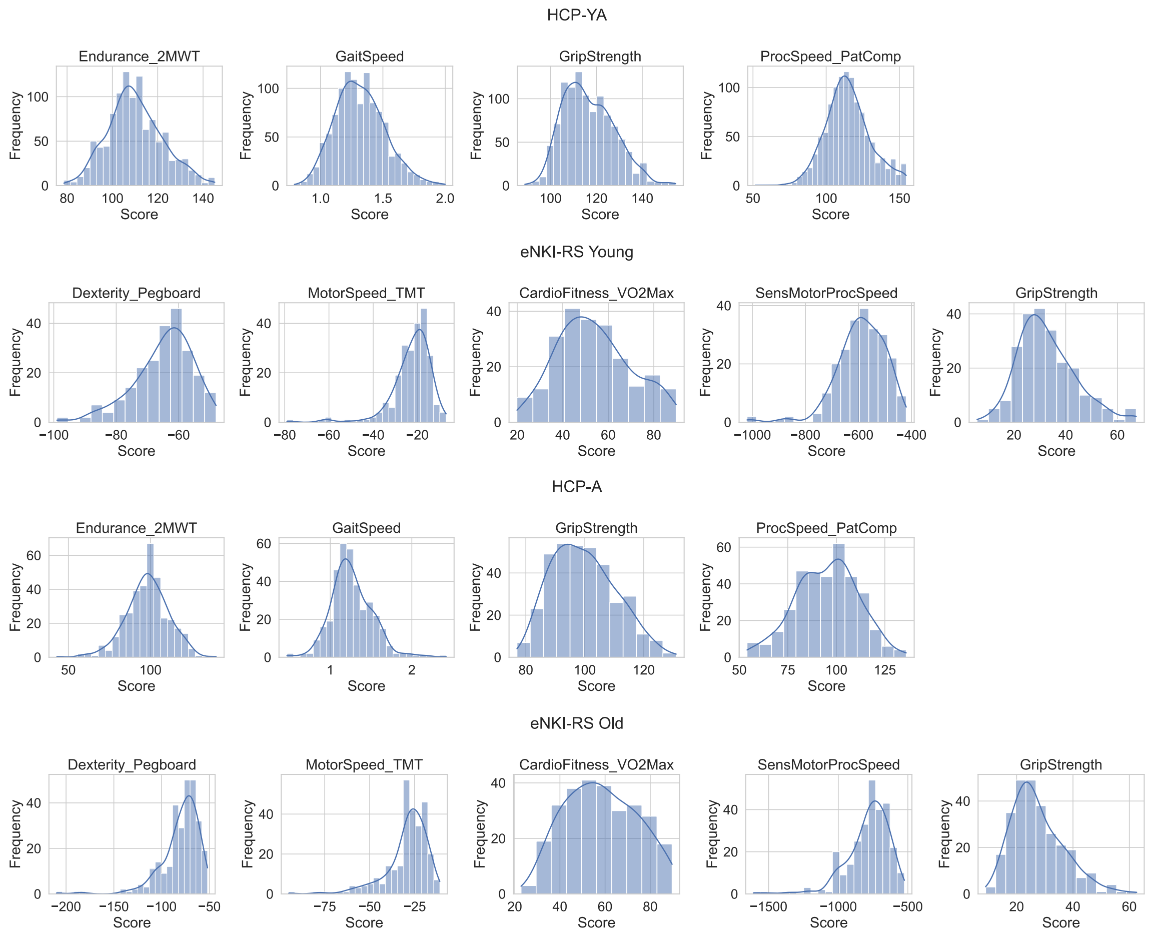

Figure S11. Distribution of the motor-performance items. Items from the HCP-YA and HCP-A show age unadjusted norm scores as provided by the Human Connectome Project. The remaining scores show raw scores in seconds (MotorSpeed_TMT, Dexterity_Pegboard), milliseconds (SensMotorProcSpeed), kilograms (GripStrength) and per millilitres per kilogram per minute (predicted VO2max). Some scores were reversed so that higher scores signify better performance.

References

1. Bookheimer, S. Y. *et al.* The Lifespan Human Connectome Project in Aging: An overview. *Neuroimage* **185**, 335–348 (2019).
2. Nooner, K. B. *et al.* The NKI-Rockland Sample: A Model for Accelerating the Pace of Discovery Science in Psychiatry. *Front. Neurosci.* **6**, (2012).
3. Van Essen, D. C. *et al.* The WU-Minn Human Connectome Project: An overview. *NeuroImage* **80**, 62–79 (2013).
4. Buysse, D. J., Reynolds, C. F., Monk, T. H., Berman, S. R. & Kupfer, D. J. The Pittsburgh sleep quality index: A new instrument for psychiatric practice and research. *Psychiatry Research* **28**, 193–213 (1989).
5. Achenbach, T. M. *DSM-Oriented Guide for the Achenbach System of Empirically Based Assessment (ASEBA)*. (ASEBA, Burlington, Vermont, 2013).
6. Achenbach, T. M. & Rescorla, L. Manual for the ASEBA adult forms & profiles. (2003).
7. Reuben, D. B. *et al.* Motor assessment using the NIH Toolbox. *Neurology* **80**, S65–S75 (2013).
8. Weintraub, S. *et al.* Cognition assessment using the NIH Toolbox. *Neurology* **80**, (2013).
9. Åstrand, P.-O. & Ryhming, I. *A Nomogram for Calculation of Aerobic Capacity (Physical Fitness) From Pulse Rate During Submaximal Work*. *Journal of Applied Physiology* **7**, 218–221 (1954).
10. Delis, D. C., Kaplan, E. & Kramer, J. H. Delis-Kaplan executive function system. *Assessment* (2001).
11. Gur, R. Computerized Neurocognitive Scanning: I. Methodology and Validation in Healthy People. *Neuropsychopharmacology* **25**, 766–776 (2001).
12. Reitan, R. M. *Trail Making Test : Manual for Administration and Scoring*. (Reitan Neuropsychology Laboratory, Tucson, Ariz, 1992).
13. Achenbach, T. M., Bernstein, A. & Dumenci, L. DSM-Oriented Scales and Statistically Based Syndromes for Ages 18 to 59: Linking Taxonomic Paradigms to Facilitate Multitaxonomic Approaches. *Journal of Personality Assessment* **84**, 49–63 (2005).
